# Alcoholic beer fermentation: impact of glutathione and peroxidase fortification on deoxynivalenol reduction

**DOI:** 10.1007/s12550-026-00674-w

**Published:** 2026-08-24

**Authors:** Danilo Salustiano dos Santos, Larine Kupski, Anderson Ferreira Vilela, Eliana Badiale-Furlong, Jaqueline Garda-Buffon

**Affiliations:** 1 School of Food Chemistry, Federal University of Rio Grande (FURG), Rio Grande, Rio Grande do Sul Brazil; 2 Department of Agroindustrial Management and Technology, Federal University of Paraíba (UFPB), Bananeiras, Paraíba Brazil

**Keywords:** Fermentation process, *Saccharomyces cerevisiae*, Protective molecules, Adsorption, Mycotoxin

## Abstract

The use of contaminated cereals in fermentation processes can elicit a marked response from yeasts in the presence of mycotoxins, as indicated by the production of defense compounds such as peroxidase (PO) and glutathione (GSH) to reduce mycotoxin damage. The objective of this study was to investigate the effect of fortifying GSH and PO levels in alcoholic fermentation as a strategy for reduce concentration deoxynivalenol (DON) during the brewing process. The QuEChERS method was validated for quantifying the mycotoxin and estimating reduction. Fermentation was conducted in a reactor containing 20 mL of synthetic wort inoculated *with Saccharomyces cerevisiae* (0.115 g). The reduction of DON was evaluated using a 2^4−1^ fractional factorial design with the following independent variables: GSH concentration (0–50 mg L^-1^), PO activity (0–0.4 U mL^-1^), fortification time (0–48 h), and DON concentration (0.1–0.3 µg mL^-1^). The results of reducing the concentration of DON in experimental planning ranged from 76,2% to non-detection of mycotoxin, significantly, with PO activity as a significant variable. Fortification with GSH and PO was significant in the decrease of DON when it was at a higher level, increasing by 39% compared to the control. Supplementation with GSH and PO demonstrated in this study indicates the effectiveness of DON reduction in the evaluated contamination levels.

## Introduction

Malted cereals are prone to fungal contamination due to their high carbohydrate content (Cui et al. 2022). Toxigenic fungi contamination can produce secondary metabolites, known as mycotoxins (Pascari et al. 2018). Due to the social, public health, and environmental impact associated with mycotoxins, special attention should be given to these contaminants through risk assessments that support the establishment of regulatory limits to prevent or minimize their toxic effects (Long et al. 2025).

Among the mycotoxins frequently detected in cereal grains is deoxynivalenol (DON), which belongs to the trichothecene group (Hooft and Bureau 2021). *Fusarium* species mainly produce them and are responsible for adverse effects on consumer health (Afacan et al. 2025; Kuzdraliński et al. 2013). DON can appear at the end of the beer production process when the grain is contaminated and transferred to the product (Mastanjevic et al. 2018). It has been demonstrated that the operations employed in processing are insufficient for the complete decontamination of DON (Mastanjevic et al. 2018). In response, strategies for mitigating these compounds have been studied, including chemical, physical and biological approaches (Afsah-Hejri et al. 2010). The recommendation is that mitigation approaches should not interfere with the sensory characteristics of the product, which has highlighted biological methods using microorganisms as a friendly alternative for mitigating contaminants such as DON (Santos et al. 2023).

Specifically in beer production, according to the literature, the first stage of the malting process (washing during the immersion process) is responsible for significantly reducing the concentration of DON (Biehl et al. 2024). This reduction is also observed in the fermentation stage, which is the main point where a reduction in mycotoxin levels is expected, resulting in a final product with less contamination than the original material (Giacomini et al. 2023).

This reduction is a consequence of the activity of specific molecules produced by yeast, highlighting the tripeptide glutathione (GSH) and the enzyme peroxidase (PO), which also participate in various cellular physiological processes (Boeira et al. 2021; Machado et al. 2022). Among these, the biotransformation of xenobiotics, acting as cofactors in reactions, maintains normal biological functions (Dobritzsch et al. 2020; Pastore et al. 2003), oxidation of phenolic structures (Ding et al. 2020), and cellular defense mechanisms with the potential to promote DON mitigation (Garcia et al. 2018; Pelyhe et al. 2018; Staerck et al. 2017). In addition to classical fermentation parameters, recent evidence suggests that peroxidase-like activities and the glutathione-based antioxidant and detoxification system, which involves reduced glutathione (GSH) and the glutathione S-transferase (GST) enzyme family, may participate in the biotransformation of mycotoxins in beer through oxidation and conjugation pathways that reduce their reactivity and bioavailability. This mechanistic hypothesis is supported by recent bioprocess studies (Liu et al. 2024; Ding et al. 2023; Kłosowski et al. 2023; Wangsanut and Pongpom 2022; Pascari et al. 2018; Freire et al. 2019).

This context suggests that adding these molecules to the process could increase the reduction efficiency of alcoholic fermentation, possibly adsorption and/or degradation related mechanisms. However, reports considering this possibility are scarce in scientific literature. Therefore, the objective of this study was to investigate the effect of fortifying GSH and PO levels in alcoholic fermentation as a strategy for reduce DON levels in the brewing process.

## Materials and methods

### Materials

The beer wort (Dry Brew brand powdered malt extract) and high fermentation *Saccharomyces cerevisiae* UC-05 yeast were purchased from a brewing supplies company in Porto Alegre, Rio Grande do Sul, Brazil. DON mycotoxin standard, PO, and GSH were purchased from Sigma-Aldrich (Saint Louis, Missouri, USA). PO was type VI, derived from freeze-dried horseradish peroxidase (*Armoracia rusticana*) in concentrations ranging from 250 to 330 U mg^− 1^, and GSH in the form of reduced L-glutathione with purity (≥ 99%), produced in Japan.

Acetonitrile (HPLC grade) was obtained from Panreac (Spain) and ultrapure water (resistivity 18.2 MΩ•cm) was obtained by purification with a Milli-Q Water System (Direct-Q UV3) (Millipore, Bedford, MA, USA) with a membrane filter (pore diameter 0.45 μm). Other reagents used were purchased in analytical standard grade.

The DON analytical solution was prepared from 1 mg of the mycotoxin. Subsequently, the working solution (50 µg mL^− 1^**)** was dissolved in benzene: acetonitrile (95:5, v v^− 1^), a mixture selected due to the high solubility of DON in aromatic–nitrile systems, which ensures complete dissolution and stability of the stock standard. It is worth noting that benzene was used exclusively for the preparation of stock solutions and was not employed at any stage of the extraction procedure. All stock concentrations were verified using a UV–Vis spectrophotometer (FEMTO Cirrus-80), according to AOAC recommendations, based on the molar absorptivity of DON in acetonitrile at 220 nm (ε = 6808) (AOAC 2000; Truckless et al. 2010). The solutions were stored at − 4 °C.

The PO standard (250 to 330 U mg^− 1^) was solubilized in sodium phosphate buffer (0.1 M, pH 6.0) to enhance enzyme activity stability, with the activity of the stock solution measured before each assay to achieve the desired activity. The GSH solution was prepared from 0.01 g dissolved in 0.01 M HCl to a volume of 100 mL (kept covered with aluminum foil to prevent oxidetion at -4 °C).

## Methods

### Preparation of beer wort and determination of physical and chemical characteristics

The synthetic wort was prepared according to the manufacturer’s instructions, using 14.28 g of powdered malt extract to prepare 100 mL of wort with sterile water. Characterization was performed according to the Association of Official Analytical Chemists International AOAC (2000) in relation to pH, ash (nº. 923.03), proteins by the Kjeldahl method (nº. 992.93), total acidity (nº. 947.05) and total soluble solids (nº. 932.12). Lipids (Folch et al. 1957), apparent density (Bueno and Degreve 1980), quantification of non-reducing sugars (Zoecklein et al. 2001) and reducing sugars by the spectrophotometric method using 3,5-dinitrosalicylic acid (DNS) (Miller 1959) were also evaluated.

### Effect of GSH and PO fortification on alcoholic fermentation

The variables PO concentration, GSH concentration, PO enzyme activity in the fermentation medium, and GSH and PO fortification time were evaluated using a 2^4−1^ fractional experimental design with 3 central points, totaling 11 experiments as described in Table 1.

**Table 1:** Variables used in the study for DON reduction in alcoholic fermentation by fractional factorial design 2^4−1^

Parameters	Levels		
	-1	0	+ 1
DON (µg mL^-1^)	0.1	0.2	0.3
GSH (mg L^-1^)	0	25.0	50.0
PO (U mL^-1^)	0	0.2	0.4
Fortification Time (h)	0	24	48

*DON* deoxynivalenol, *GSH* glutathione, *PO* peroxidase, *h* hour

The selected PO activities (0–0.4 U mL⁻¹) were based on values previously reported for mycotoxin reduction using oxidative enzymes (Garcia et al. 2020; Sibaja et al. 2018; Machado et al. 2022). GSH concentrations (0–50 mg L⁻¹) were established considering preliminary observations and physiological levels previously observed during beer fermentation in our laboratory, since studies evaluating extracellular GSH fortification for DON reduction are currently unavailable.

The alcoholic fermentation experiment was conducted in 50 mL reactors sterilized in an autoclave for 15 min at 121 °C. In the reactor, the mycotoxin concentration corresponded to the experimental design. The solvent from the standard solution was evaporated under a nitrogen stream that permeated through a 0.22 μm filter. Afterwards, 2 mL of wort was added, stirred in an ultrasonic bath (25 kHz) for 3 min and then vortexed for 2 min. The mixture was left to stand for 24 h prior to use.

After 24 h, the yeast (0.115 g) was added to the reactor containing beer wort and adjusted to 20 mL. The medium was kept in a germination chamber at 19 °C for 96 h (without agitation).

To evaluate DON reduction, the addition of a GSH volume or enzyme solution corresponding to the planning in Table 1 was performed. The addition was performed in order to maintain the final volume of the fermentation medium at 20 mL at the start of fermentation. At the end of fermentation, the residual concentration of DON was determined. A control test was performed under the same study conditions described for the central point of the experiment, without the addition of mycotoxin.

### Effect of fortification with GSH and PO at higher concentrations of DON

After obtaining the data through a fractional factorial design, the effect of GSH and PO fortification using DON in higher concentrations was investigated. The DON concentration was based on the level regulated by Brazilian legislation and the water: barley ratio used in beer production.

The process conditions were the same as those described in the previous item, maintaining a fermentation period of 96 h and a reactor volume of 20 mL. The following tests were performed: (I) Control, where the medium consisted of wort contaminated with DON at 2.0 µg mL^-1^ and yeast (0.115 g) and (II) Fortified, with wort contaminated with DON at 2.0 µg mL^-1^, yeast (0.115 g), fortification of GSH (25 mg L^-1^) and PO (0.2 U mL^-1^) in 24 h of fermentation. A DON concentration ten times higher than the maximum level established for barley was selected to challenge the proposed strategy under severe contamination conditions. A test without DON contamination was performed to monitor the effect of the fermentation process.

### Determination of DON in fermented media

DON extraction was performed using the QuEChERS method, as described by Arraché et al. (2018), with modifications. The modification of the method consisted of miniaturization, with reductions in the amount of sample (1 mL), acetonitrile (5 mL), extraction salts, 2 g of magnesium sulfate (MgSO_4_) and 0.5 g of sodium chloride (NaCl) and cleaning salts (0.45 g of MgSO_4_ and 0.15 g of cellite). The method was modified by miniaturizing the extraction procedure due to the limited volume of fermented samples (20 mL) obtained from each experimental reactor.

DON detection and quantification were performed at 220 nm in a liquid chromatograph (LC) equipped with a diode array detector (DAD – 10 AXL). Separation occurred in a reverse phase C_18_ column (Gemini^®^, Phenomenex) 250 × 4.5 mm (5 μm), the mobile phase consisting of MeCN and Mili-Q water (30:70, v v^− 1^), flow rate of 0.8 mL min^− 1^ and volume injection 20 µL (Souza et al. 2015). The extracts that yielded concentrations below the limit of quantification (LQ) were concentrated under a nitrogen stream up to 20 times, followed by a new chromatographic injection to estimate the reduction in DON.

After extraction, samples were concentrated prior to chromatographic analysis. Samples with no detectable DON peak (signal below the LOD) were considered as presenting 100% DON reduction for data presentation.

The reduction in DON mycotoxin (%) was estimated by the ratio between the initial concentration added to the reactor and that determined at the end of fermentation (Eq. 1), where MC is the mycotoxin concentration.

1 $$Reduction(\%)=100-\left(\frac{MC(final)\;.\;100}{MC(initial)}\right)$$

### Method validation

The analytical method for DON determination was validated according to the performance criteria established by the European Commission (2023). The evaluated parameters included linearity, coefficient of determination (R^2^), limit of detection (LOD), limit of quantification (LOQ), accuracy, and precision. The method was considered acceptable when recoveries ranged from 70 to 120%, relative standard deviation (RSD) values were below 20%, and the coefficient of determination (R^2^) exceeded 0.99.

Linearity was assessed by constructing analytical calibration curves using external standardization in solvent and matrix-matched calibration, each covering the concentration range of 0.05–5.0 µg mL^− 1^. The coefficient of determination (R^2^) was obtained by linear regression between analyte concentration and instrumental response, representing the proportion of variance explained by the regression model.

The LOD and LOQ were estimated using the signal-to-noise ratio approach, corresponding to signal intensities at least three and ten times higher than the baseline noise, respectively. Accuracy was evaluated through recovery assays performed in triplicate at three fortification levels (0.05, 0.25, and 0.50 µg mL^− 1^). Beer samples were fortified by adding a known volume of the working standard solution to 1 mL of sample. Precision was expressed as the relative standard deviation (RSD) obtained from the triplicate recovery assays.

The validation experiments were conducted under single-laboratory conditions for the analytical application proposed in this study. Therefore, intermediate precision and inter-laboratory reproducibility were not evaluated.

### Determination of GSH

To determine GSH, the colorimetric method proposed by Owens and Belcher (1965) was used, with a modification in the sample volume (1 mL) for analysis. The reaction was carried out using 5,5-dithiobis-2-nitrobenzoic acid (DTNB). After the reaction, absorbance was measured at 412 nm in a spectrophotometer (FEMTO Cirrus-80), and the GSH concentration (mg L⁻¹) was calculated using the L-GSH standard curve (0.7–9.0 mg L^− 1^).

### Determination of PO enzyme activity

PO activity was evaluated in a spectrophotometer at 470 nm, according to the manufacturer’s instructions (Sigma-Aldrich 2019), with modifications (Devaiah and Shetty 2009) using hydrogen peroxide and guaiacol as substrates, where one peroxidase unit (U) was defined as the amount of enzyme capable of oxidizing 1 µmol of substrate per minute. Enzymatic activity was estimated using Eq. (2).

2 $$\frac U{ml}\cdot\frac{Abs\;.\;V\;.\;DF\;.\;10^6}{\varepsilon\;.\;d\;.\;t\;.\;v\;.\;1000}$$

Where *Abs* is the absorbance, *ε* is the molar absorptivity of tetraguaiacol (26,600 L mol^− 1^ cm^− 1^), *V* is the total volume of the reaction tube (mL), *v* is the volume of enzyme extract used (mL), *t* is the reaction time in minutes, *d* is the cuvette size (cm), and *DF* is the dilution factor of the enzyme extract.

### Statistical analysis

All determinations related to the physical and chemical characterization of the beer wort were performed in triplicate, and the results were expressed as means and standard errors. The fractional experimental design was statistically evaluated after assessing the normality of the data using Statistica 7 software at a 10% significance level (*p* < 0.10). Student’s t-test was used to observe the statistical differences among the fermentation assays with higher DON concentrations at a 5% significance level (*p* < 0.05).

## Results and discussion

### Physical and chemical composition of beer wort

Synthetic wort powder was chosen primarily to standardize the composition of the medium, as well as to ensure that the wort was not contaminated with DON and that the experiments were reproducible, since it would serve as a laboratory model for investigating the effect of increasing GSH and PO levels on reducing DON concentration. The composition of the wort determines the flavor and aroma characteristics of the beer, where biotransformation mainly produces ethanol and carbon dioxide from the action of the yeast (Stewart 2016). To confirm that the wort presented physicochemical characteristics suitable for alcoholic fermentation the synthetic wort was characterized in this study, as shown in Table 2.

**Table 2:** Physical and chemical composition of synthetic powdered beer wort

Parameters	Triplicate averages ± Standard error
Total sugars (%*)	47.7 ± 0.59
Reducing sugars (%)	23.3 ± 0.51
Total proteins (%)	6.5 ± 0.15
Ash (%)	6.8 ± 0.80
Lipids (%)	0.9 ± 0.11
Total Acidity (mEq**L^− 1^)	24.4 ± 0.05
Soluble solids (ºBrix)	12.4 ± 0.01
pH	5.4 ± 0.01
Apparent density (ρ) (g mL^− 1^)	1.0 ± 0.01

*% - g 100 g^− 1^; **mEq L^− 1^ – milliequivalents per liter

The concentration of carbohydrates and proteins provided on the synthetic wort label was 88% and 6.8%, respectively, which is similar to the results determined in this study. The supplier also reports that there are no significant concentrations of lipids in the wort, which is in agreement with the data found in this study.

The ideal pH for beer wort is between 5.2 and 5.5, and the Brix degree is between 11 and 12. Therefore, both the hydrogen ion potential result of 5.4 and the Brix degree of 12.4 were in line with previous studies in scientific literature (Flores-Calderón et al. 2017; Piacentini et al. 2015). The total acidity and apparent density values were also in line with those reported in the literature (Wyler et al. 2015).

### Method validation

The analytical quality data for the method used in the extraction and quantification of DON, as well as the estimation of DON reduction, are presented in Table 3.

**Table 3:** Performance parameters of the analytical method used to determine DON in beer

Parameters	Results
Operating range (µg mL^− 1^)	0.05–0.5
Curve equation	y = 119970x – 167.77
R^2^	0.994
LOQ (µg mL^− 1^)	0.05
LOD (µg mL^− 1^)	0.015
Recovery (%), RSD (%)	
Level 1 (0.05 µg mL^− 1^)	105.43 (8.3)
Level 2 (0.25 µg mL^− 1^)	73.43 (12.6)
Level 3 (0.5 µg mL^− 1^)	74.06 (2.0)

*R*^2^ coefficient of determination, *LOQ* quantification limit, *LOD* detection limit, *RSD* Relative standart deviation

The method was validated to ensure reliable results (European Commission 2023). The method can generally be considered accurate, precise, and sensitive, as demonstrated by the low limits of detection (LOD) and quantification (LOQ), the recovery values, and the coefficient of determination (R^2^). The linearity of the curve met the range applied in this study, with a coefficient of determination (R^2^) of 0.994. In addition, recoveries ranging from 70 to 120% were considered satisfactory for this type of contaminant and demonstrated the accuracy of the method, while RSD values below 20% confirmed its precision (European Commission 2023). Studies by Garcia et al. (2020), Habler et al. (2017), and Rausch et al. (2021) also reported validation results similar to those obtained in the present study for DON determination in beer.

Therefore, the proposed method proved to be reliable for monitoring DON reduction during beer fermentation, in accordance with the performance criteria established by the European Commission (2023).

### DON reduction by fortification with GSH and PO

The coded and actual values at the levels used in the design, together with the reduction results obtained for the mycotoxin DON, expressed as a percentage, are shown in Table 4.

**Table 4:** DON reduction (%) in alcoholic fermentation with *S. cerevisiae* with PO and GSH fortification evaluated by fractional factorial design 2^4−1^

Assays	Coded Values					Real Values					Answer
	X1 Peroxidase	X2 Glutathione	X3 DON		X4 Time	X1 Peroxidase (U mL^− 1^)	X2 Glutathione (mg L^− 1^)	X3 DON (µg mL^− 1^)		X4 Time (h)	Reduction (%)
1	-1	-1	-1		-1	0	0	0.1		0	100
2	1	-1	-1		1	0.4	0	0.1		48	83.6
3	-1	1	-1		1	0	50	0.1		48	94.2
4	1	1	-1		-1	0.4	50	0.1		0	72.6
5	-1	-1	1		1	0	0	0.3		48	95.1
6	1	-1	1		-1	0.4	0	0.3		0	86.5
7	-1	1	1		-1	0	50	0.3		0	82.5
8	1	1	1		1	0.4	50	0.3		48	93.2
9	0	0	0		0	0.2	25	0.2		24	86.3
10	0	0	0		0	0.2	25	0.2		24	88.1
11	0	0	0		0	0.2	25	0.2		24	87.2

*DON* Deoxynivalenol, *h* hour

The studied variables were chosen based on previous studies in the literature. Fortification with GSH and PO at 0, 24, and 48 h of fermentation was used. Previous studies have reported that the stationary phase begins after approximately 48 h of fermentation, a stage generally associated with increased cellular stress due to reduced substrate availability and the presence of inhibitory compounds such as mycotoxins (Boeira et al. 2021; Dong et al. 2007). There is no specific legislation establishing a maximum limit for DON in beer. Therefore, the DON concentrations evaluated (0.1–0.3 µg mL^-1^) were selected based on previous studies involving alcoholic fermentation (Boeira et al. 2021; Garda et al. 2005).

DON reduction during the fermentation process varied among the experimental assays, with reductions ranging from 72.6% to the absence of a detectable DON peak under the chromatographic conditions employed in one assay. The highest reduction value (100%) corresponded to an assay in which no DON chromatographic peak was detected after fermentation. According to the criteria adopted in this study, this condition was considered as 100% DON reduction. These results allowed the effects of the studied variables to be evaluated using the 2^4−1^ fractional factorial design (Table 5).

**Table 5:** Effects generated by fractional factorial design 2^4−1^ by fortification of GSH levels (mg L^− 1^) and PO activity (U mL^− 1^) on DON reduction (%) in beer fermentation

Variable	Effect	Standard Deviation	t (6)	Significance level (*p* < 0,10)	Coefficient
Average/Interaction	88.42000	1.955666	45.21222	0.000000	88.42000
PO	-8.94500	4.586443	-1.95031	0.099016	-4.47250
GSH	-5.62000	4.586443	-1.22535	0.266357	-2.81000
DON	1.74500	4.586443	0.38047	0.716703	0.87250
Time	6.11000	4.586443	1.33219	0.231163	3.05500

The overall average DON reduction observed in the 2^4−1^ fractional factorial design was 88.4%, considering a 10% significance level. Among the evaluated variables, only peroxidase (PO) activity showed a significant effect on DON reduction during the fermentation process, with a negative effect of − 8.9%. Thus, increasing PO activity from the low level (-1) to the high level (+ 1) decreased DON reduction by 8.9%. However, the present study did not evaluate the mechanisms involved in this response. The variables GSH concentration, initial DON concentration, and fortification time showed no significant effect on DON reduction at the 10% significance level.

After 96 h, the central points (assays 9, 10, and 11) reached an average reduction of 87.2%. These assays were fortified with 25 mg L^− 1^ of GSH in 24 h, with an enzymatic activity of 0.2 U mL^− 1^ at a DON concentration of 0.2 µg mL^− 1^. A comparable result was reported by Su et al. (2024), who achieved approximately 82.7% DON degradation after 24 h at 30 °C using a chimeric manganese peroxidase (0.1 U mL^− 1^)/glutathione system. Although the experimental systems were different, both studies indicate that enzyme-based approaches may contribute to reducing DON levels under controlled conditions.

Assays 2, 4, 6, and 7 showed reductions of 83.6, 72.6, 86.5, and 82.5%, respectively. The lowest reduction result was observed in assay 4, which was intriguingly fortified at the beginning of fermentation with the highest levels of PO and GSH at a DON concentration of 0.1 µg mL^− 1^. This result may be related to several factors, including the possible influence of initial addition of the two biomolecules on the interaction between DON and the fermentation system, although the mechanisms involved remain unclear. Other factors may also have contributed, such as variations in yeast metabolism, competition between molecules, the high GSH concentration, and modifications in the enzyme active site caused by the pH conditions (Garcia et al. 2020). Additionally, the action of GSH may be compromised by time, temperature and oxidation-reduction reactions (Junior et al. 2001).

**Fig. 1:**
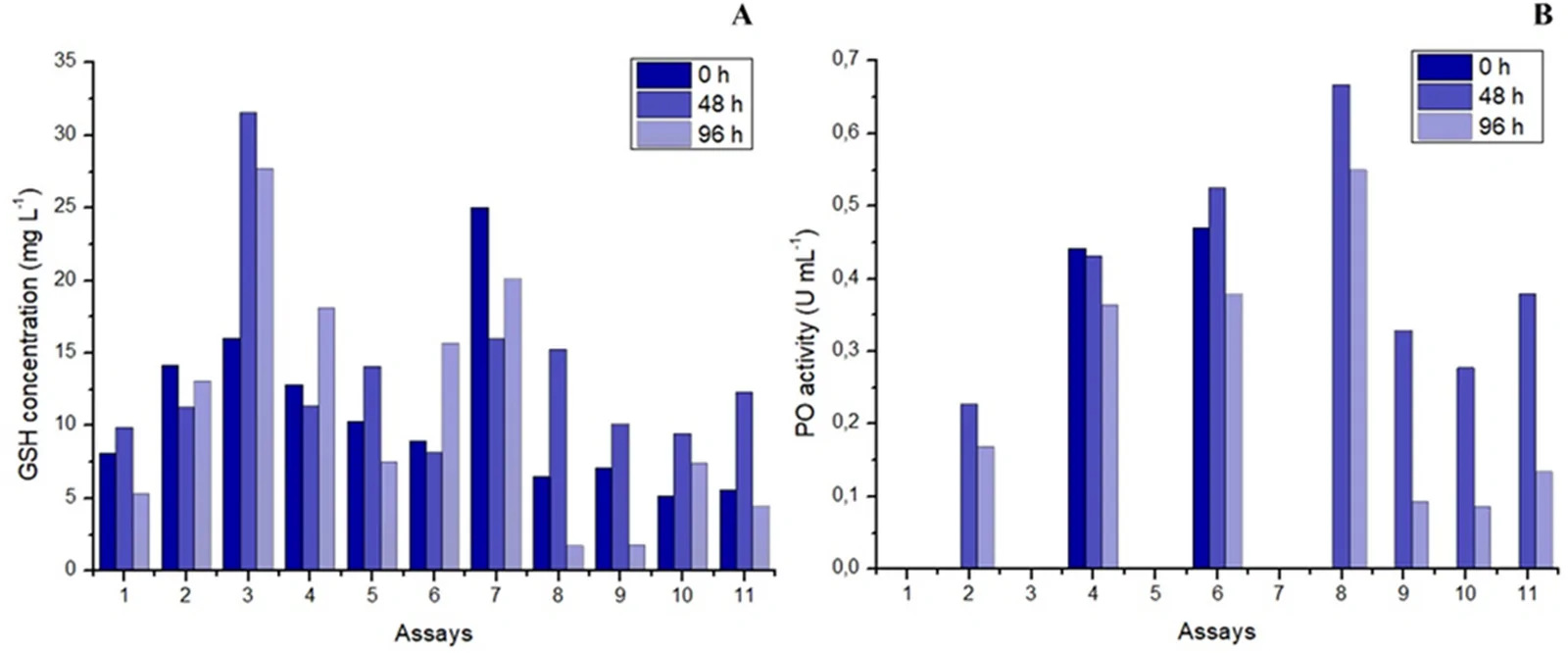
GSH concentration (**A**) and peroxidase (PO) activity (**B**) during beer alcoholic fermentation under the experimental conditions of the 2⁴⁻¹ fractional factorial design, evaluated at 0, 48, and 96 h of fermentation. Assays 1–8 correspond to single experimental runs, each representing a distinct combination of the evaluated variables according to the experimental design. Assays 9–11 represent the replicated center points and were performed in triplicate to estimate the pure experimental error and evaluate the curvature of the model. For the center-point assays, GSH concentrations (mg L^− 1^) were 5.92 ± 0.98 (0 h), 10.61 ± 1.50 (48 h), and 4.55 ± 2.83 (96 h), whereas PO activities (U mL^− 1^) were 0.07 ± 0.04 (0 h), 0.33 ± 0.05 (48 h), and 0.10 ± 0.03 (96 h), expressed as mean ± standard deviation

It should be noted that at the highest percentages of DON reduction occurred in trials 1 (100%) and 5 (95.1%), with the common characteristic of non-fortification with GSH and PO, differing only in DON concentration (0.1 and 0.3 µg mL⁻¹). Another mechanism possibly associated with DON reduction during alcoholic fermentation is physical adsorption onto yeast cell wall components, in which the mycotoxin content adheres to the yeast cell wall due to the presence of nanoproteins and β-glucans, as suggested by the study conducted by Wall-Martínez et al. (2019). Considering this possibility, GSH levels and PO activity were determined in the fermented material, as shown in Fig. 1.

Figure 1A shows that the GSH concentration varied across all assays, with the highest concentration observed in the fortified ones. Although assays 1 and 7 differed in more than one experimental factor, a comparison between them suggests that GSH fortification was not associated with higher DON reduction under the conditions evaluated in the present study. Assay 7, fortified with 50 mg L^− 1^ of GSH, showed no detectable DON peak at the end of fermentation. This outcome may be related to the multiples roles of GSH, such as regulating apoptotic pathways, maintaining cellular homeostasis and redox balance, and modulating the immune system, which may have limited its effectiveness in detoxifying the xenobiotic (DON) (Hu et al. 2025). Regarding the PO enzyme, Fig. 1B shows that only the fortified assays exhibited detectable enzymatic activity, which is consistent with the hypothesis that a physical adsorption to yeast cell wall components may have contributed, at least partially, to the observed reduction of DON.

Therefore, based on the experimental design and the conditions evaluated in the present study, the assays without GSH and PO fortification showed the highest DON reductions. To investigate whether these biomolecules would exert any effect under higher DON concentrations, an additional experiment was conducted.

### Effect of fortification with GSH and PO on DON concentration higher than 2 µg mL^− 1^

**Fig. 2:**
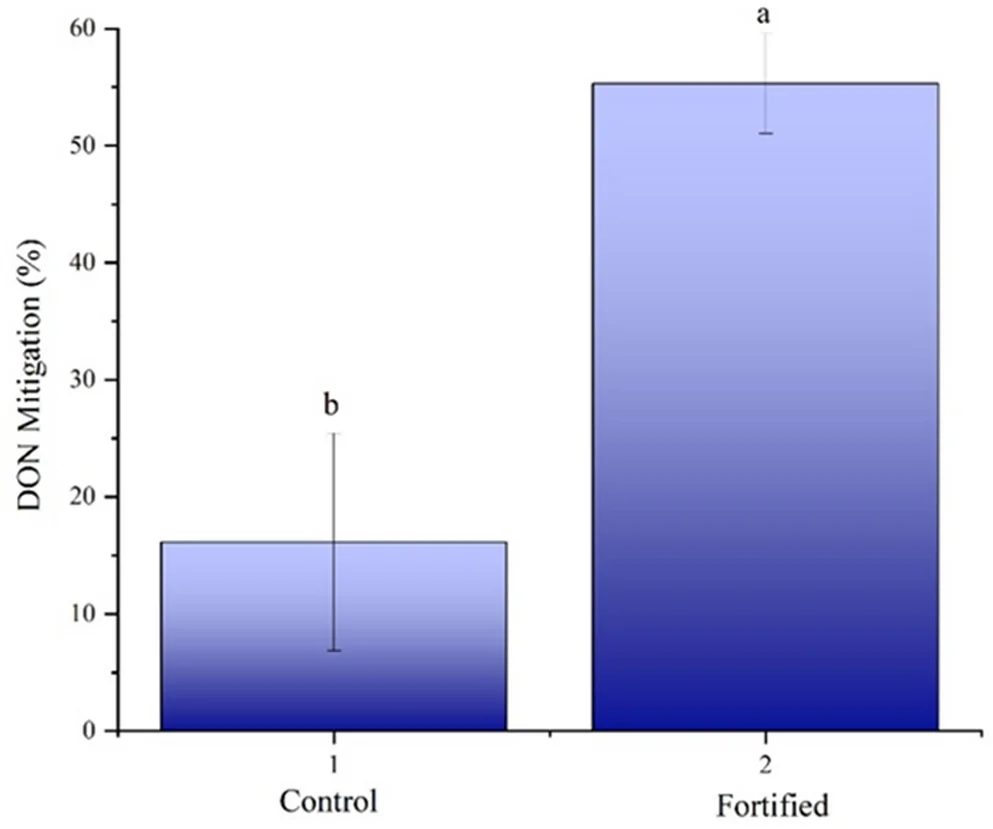
DON reduction (%) in beer alcoholic fermentation with and without fortification of GSH levels (mg L^− 1^) and PO enzyme activity (U mL^− 1^). Different letters indicate statistically significant differences as determined by Student’s t-test at a significance level of 5% (*p* < 0.05)

To investigate the potential of yeast in DON reduction, a fermentation medium contaminated with 2 µg mL^− 1^ of DON was employed, concentration selected as a stress condition model to evaluated the maximum reduction capacity under elevated DON contamination. The experiment involved fortifying of the medium after 24 h with GSH (25 mg L^− 1^) and PO (0.2 U mL^− 1^), following the central points of the previous experimental design, and was compared with a non-fortified control (Fig. 2).

Mycotoxin reduction in the control assay was 16.7%, showing a significant difference (*p* > 0.05) compared to the fortified assays with GSH and PO, which achieved a reduction of 55.3%, 38.6% more than the control. These results suggest that, under the experimental conditions evaluated, yeast alone was less effective in reducing DON at the higher contamination level, whereas GSH and PO fortification was associated with a greater reduction of the mycotoxin.

The efficiency of PO in reducing mycotoxin levels in the fermentation process has already been described in the literature with positive results, as seen in the study by Garcia et al. (2020), which showed a simultaneous reduction of OTA and zearalenone levels of 27 and 64.9%, respectively, in a model beer solution, Liu et al. (2023) also investigated the application of three recombinant peroxidases as a degrading agent for aflatoxin M1 in a model beer solution, achieving mitigation results of up to 73.9%.

During the beer fermentation process, multiple microbial metabolic pathways are activated to maintain redox balance and cellular homeostasis. These pathways include enzymes with oxidative and conjugative functions, which can indirectly act in the biotransformation or removal of mycotoxins from the medium. Among these are peroxidases and the glutathione (GSH/GST) system, which have been associated with the attenuation of electrophilic compounds and xenobiotics in various bioprocesses (Liu et al. 2024; Ding et al. 2023; Kłosowski et al. 2023).

Under typical alcoholic fermentation conditions, pH variations (4.0–5.0) and the presence of reactive oxygen species (ROS), generated by residual respiration or the oxidation of wort phenols, create a favorable environment for the activity of peroxidases. These enzymes catalyze oxidation reactions involving aromatic substrates, potentially generating intermediates that may alter mycotoxin reactivity (Liu et al. 2024; Loi et al. 2023).

Recent studies have demonstrated that peroxidases can degrade aflatoxin M1 and AFB1 in liquid matrices, such as milk and beer, without compromising the sensory properties of the final product (Liu et al. 2023; Ding et al. 2023), thereby reinforcing the biotechnological potential of this mechanism in fermentation systems. The study by Sibaja et al. (2018) demonstrated the effectiveness of PO (0.015 U mL⁻¹) in mitigating the mycotoxins AFB1 and AFM1 in milk and beer, achieving reductions of 97% and 24% for AFB1 in milk and beer, respectively, and 65% for AFM1 in milk. Similar mitigation results due to the action of PO (0.015 U mL⁻¹) were also reported in the study by Kerstner et al. (2024), reaching 78.2% and 71.2% in milk for the mycotoxins AFB1 and AFM1, respectively.

Concurrently, the glutathione (GSH) system serves as an important endogenous pathway for antioxidant protection and detoxification in *Saccharomyces cerevisiae*. Glutathione S-transferase (GST) catalyzes conjugation reactions between the sulfhydryl group of GSH and electrophilic xenobiotics, possibly altering the reactivity and polarity of mycotoxins, which may favor interactions with cellular structures or sequestration in the fermentation medium (Wangsanut and Pongpom 2022; Mariani et al. 2008). This process is regulated by fermentation conditions, oxygen limitation, nutrient gradient and increased ethanol concentration are factors that modulate the expression of GSTs and glutathione peroxidase (Kho et al. 2006; Kłosowski et al. 2023). The integration of peroxidase oxidation and conjugation via GSH/GST pathways may support the hypothesis of a sequential mechanism: initially, partial oxidation of mycotoxins occurs by peroxidases, followed by conjugation or sequestration interactions involving GSH and cell wall components. This hypothesis is supported by previous studies reporting the formation of modified metabolites or conjugates of mycotoxins during alcoholic fermentations, such as less toxic OTA and ZEA derivatives (Freire et al. 2019; Pascari et al. 2018). Conjugation and possibly subsequent co-sedimentation of the toxin with yeast biomass may partially explain the observed reductions in the soluble fraction of fermented beverages, even in the absence of complete degradation of the original chemical structure.

In the study by Giacomini et al. (2023), after 168 h of alcoholic fermentation with *Sacchoromyces cerevisiae* (10 g L^− 1^), a 58% reduction of the mycotoxin ochratoxin A (OTA) was observed. The authors highlighted that the reduction in this contaminant was directly related to metabolic processes in the yeast cells, mainly PO activity and GSH production.

Furthermore, operational parameters such as fermentation temperature, pH, and yeast flocculation intensity can impact the efficiency of these pathways. Yeasts with high flocculation and higher intracellular GSH accumulation tend to have higher toxin sequestration capacity, while strains with lower intracellular redox potential may exhibit lower detoxification efficiency. The use of adjuncts rich in phenols and reducing compounds can also favor peroxidase-mediated reactions, enhancing mycotoxin removal (Lee et al. 2024; Ding et al. 2023).

Taken together, these results suggest that alcoholic fermentation may act as a bioprocess associated with the reduction of detectable mycotoxin levels through oxidative and conjugative pathways. Therefore, the fortification strategy evaluated in this study may represent a promising biotechnological approach for partially mitigating mycotoxins in fermented beverages and the application of biomolecules, such as PO and GSH, is promising for mitigating mycotoxins in the food industry, primarily due to the specificity of each molecule (Ji et al. 2016; Raveendran et al. 2018). The results obtained in the present study suggest that PO fortification, together with GSH supplementation, may contribute to increased DON reduction under the higher contamination level evaluated (2 µg mL^− 1^).

This study investigated the effect of GSH and PO fortification on DON reduction during the fermentation step of beer production using a fractional factorial design and obtained DON reductions ranging from 72.6 to 100%.

At low DON levels (0.1 to 0.3 µg mL^− 1^), the addition of GSH and PO did not significantly change the ability of *S. cerevisiae* to reduce DON, which may be related to adsorption of the mycotoxin in the cell wall. However, at a level of 2 µg mL^− 1^, GSH and PO were associated with a 39% increase in DON reduction, suggesting that additional mechanisms may be involved in DON reduction at higher contamination levels and contribute to reducing detectable DON levels in fermented beverages. The fortification strategy evaluated in this study may represent a promising alternative for reducing DON levels during alcoholic fermentation. However, additional studies evaluating fermentation performance, yeast physiology and DON biotransformation products are necessary to better understand the mechanisms involved and to assess the applicability of this strategy under industrial conditions.

## Data Availability

No datasets were generated or analysed during the current study.

## Abbreviations

DON: Deoxynivalenol
PO: Peroxidase
GSH: Glutathione
GST: Gluthatione S-transferase
